# Polarized upconversion emission at metasurface

**DOI:** 10.1038/s41377-023-01301-4

**Published:** 2023-11-06

**Authors:** Zhichao Yang, Dayong Jin

**Affiliations:** Institute for Biomedical Materials & Devices, Faculty of Science, University of Technology Sydney, Ultimo, NSW 2007 Australia

**Keywords:** Metamaterials, Nanocavities

## Abstract

Leveraging the resonant modes of all-dielectric metasurfaces, specifically quasi-bound state in the continuum and Mie resonances, the precise orthogonal polarization control has been realized.

Upconversion luminescence is a distinctive phenomenon, eliciting widespread attention due to its intrinsic optical properties and diverse applications. This phenomenon involves material transitions from lower-energy states to higher-energy states, releasing photons with energies exceeding those of the absorbed photons. Particularly, for sensing, microscopy, bioimaging, communication, and photon energy management, upconversion luminescence offers capabilities to realize solutions as versatile fluorescent nanoscale emitters and energy transducers^1^. In nanophotonics, enhancing upconversion luminescence and controlling their emission properties, including colours, lifetimes, and polarization, represent pivotal pursuits^2^. Stringent polarization control will enable versatile options for diverse optical implementations. However, the simultaneous attainment of fluorescence from multiple emitters (doped lanthanide ions) into tailored polarization poses a formidable challenge.

Positioning nanoparticles in a photonic cavity with the resonant modes can tune the materials’ optical properties, therefore, to yield transformative new properties and enable innovative optical device designs. In the pursuit, resonant dielectric metasurfaces have emerged as a solution with profound interests. Engineered from periodically arranged nanoscale constituents, metasurfaces provide a platform for meticulously tailored optical functionalities. In contrast to traditional optical materials and cavities, resonant dielectric metasurfaces offer an expanded degree of design freedom, enabling exquisite manipulation of light fields. Precise control over geometric parameters of these metasurfaces engenders the excitation of specific resonance modes, thus amplifying local field strengths and ushering in fresh strategies for fluorescence enhancement and polarization control^3,4^.

Presented in a recent report, published in *eLight*, Li’s research team and collaborators^5^ has achieved dual-band enhanced emissions from erbium doped upconversion nanoparticles with controlled directional polarization, through the precision design and fabrication of a type of resonant dielectric metasurface. This work foreshadows transformative impacts within the realm of investigating the interplays between the advanced design of optical metasurfaces and fluorescence upconversion nanoparticles^6,7^.

Unlike previous studies that focused on overall fluorescence polarization control, this research achieves selective polarization modulation of dual-band visible upconversion photoluminescence without the need for specific polarization excitation. This is made possible using resonant dielectric metasurfaces, which introduce two pivotal resonance modes: the Quasi-Bound State in the Continuum (QBIC) and Mie resonance modes. These modes provide simultaneous high-Q and selective polarization resonances, enabling the efficient utilization of both QBIC and Mie resonances to achieve high enhancement factors and polarization controllability. This approach is based on a wavelength-matching strategy that can be extended to achieve more sophisticated spectral control of multi-color upconversion photoluminescence.

QBIC, rises when two leaky modes with similar far-field profiles at similar frequencies undergo avoided resonance crossing, resulting in one of the modes exhibiting an extraordinarily high-quality factor (Q-factor)^3^. By meticulously tuning structural parameters, QBIC can be harnessed on resonant dielectric metasurfaces. The high Q-factor allows QBIC modes to accumulate optical energy locally with minimal loss, significantly enhancing upconversion emission efficiency (Fig. 1 green pattern). In addition, tailoring the geometric shape and parameters of resonant dielectric metasurfaces enables the generation of distinct Mie resonance modes (Fig. 1 red pattern), arising at specific frequencies in material micro- or nanostructures. These modes confine optical energy efficiently and generate strong localized electric field enhancement, consequently amplifying upconversion emission brightness.

**Fig. 1:**
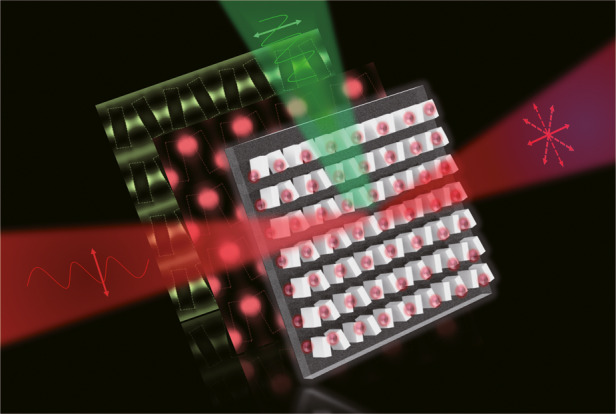
Upconversion emission enhancement with polarization control. A 980 nm laser beam is directed into a non-rotationally symmetric arrangement of nanobricks comprising an all-dielectric metasurface. Through the interplay of QBIC resonance (green pattern) and Mie resonance effects (red pattern), upconversion nanoparticles (pink spots) coated onto the surface of the nanobricks exhibit enhanced fluorescence emission with directional polarization controls

In this study, the authors harnessed both resonance modes to intensify the electric near fields around resonators. Specifically, the use of these modes can effectively enhance the electric near field with a theoretical intensity enhancement factor towards 1600, and achieve the highest reported levels^8,9^ of degree of polarization (DoP) for upconversion luminescence, reaching approximately 0.86 and 0.91 for emissions at 540 and 660 nm, respectively. One noteworthy aspect of this study is the significant enhancement of upconversion photoluminescence decay rate and the narrowing of the spectral linewidth achieved by combining resonant dielectric metasurfaces (Fig. 1).

Advancing the previous works reported by Wu^10^ and Zeng^11^, towards the same direction, this report involved in the meticulous design of nanoantenna arrays utilizing surface plasmon resonance to achieve the desirable outcomes. While the current studies exhibit their strengths, they share certain limitations, such as the demanding requirements for precise fabrication and the intricate optical designs.

Despite the remarkable progress achieved in the field of emission enhancement and polarization control, several challenges remain to be addressed. The adaptability to multiple wavelengths and fluorescence materials, as current research largely focuses on specific wavelengths for upconversion emission enhancement and polarization control. A broad range of applications demand resonant metasurfaces that can operate across diverse spectral ranges and accommodate various types of fluorophores. This necessitates further investigations to design metasurfaces applicable to a broad spectrum. Current report predominantly concentrates on linear and cross-polarization control. Yet, applications such as optical communication and quantum information processing may demand more intricate polarization states like circular polarization. Therefore, future research may include the designs for resonant dielectric metasurfaces capable of achieving complex polarization states. Since this report has resulted in the improved spectral resolution (narrowing), it might be possible to demonstrate the potential of upconversion polarization lasing if highly-doped upconversion nanoparticles may be used^12^.

In nanophotonics, where light and matter intricately intertwine, the present study reveals a novel avenue to harness this interaction through meticulously crafted resonant dielectric metasurfaces. This achievement not only addresses inherent coupling phenomena but also unlocks the potential to reshape upconversion emission. These advances permeate various applications, from versatile biological imaging to multifunctional optical devices, the journey into uncharted territories of optical innovation is both remarkable and promising.

## References

[1] Qin X (2021). Surface plasmon-photon coupling in lanthanide-doped nanoparticles. J. Phys. Chem. Lett..

[2] Wen SH (2018). Advances in highly doped upconversion nanoparticles. Nat. Commun..

[3] Zubyuk V (2021). Resonant dielectric metasurfaces in strong optical fields. APL Mater..

[4] Ding F, Tang SW, Bozhevolnyi SI (2021). Recent advances in polarization‐encoded optical metasurfaces. Adv. Photonics Res..

[5] Feng ZW (2023). Dual-band polarized upconversion photoluminescence enhanced by resonant dielectric metasurfaces. eLight.

[6] Tripathi A (2023). Metasurface-controlled photonic rashba effect for upconversion photoluminescence. Nano Lett..

[7] Tripathi A (2020). Topological nanophotonics for photoluminescence control. Nanophotonics.

[8] He, J. J. et al. Plasmonic enhancement and polarization dependence of nonlinear upconversion emissions from single gold nanorod@SiO_2_@CaF_2_:Yb^3+^, Er^3+^ hybrid core-shell-satellite nanostructures. *Light Sci. Appl.* **6**, e16217, 10.1038/lsa.2016.217 (2017). 10.1038/lsa.2016.217 PMC6062198 30167245

[9] Barreda Á (2022). Applications of hybrid metal‐dielectric nanostructures: state of the art. Adv. Photonics Res..

[10] Pan CD (2022). Angularly anisotropic tunability of upconversion luminescence by tuning plasmonic local-field responses in gold nanorods antennae with different configurations. Nanophotonics.

[11] Chen LX (2018). Selective polarization modification of upconversion luminescence of NaYF_4_:Yb^3+^, Er^3+^ nanoparticles by plasmonic nanoantenna arrays. J. Phys. Chem. C..

[12] Shang YF (2020). Low threshold lasing emissions from a single upconversion nanocrystal. Nat. Commun..

